# Are Healthcare Organizations Healthy Work Ecosystems? Health and Well-Being of Health Professionals

**DOI:** 10.3390/healthcare12222277

**Published:** 2024-11-14

**Authors:** Tânia Gaspar, Barbara Sousa, Elisabete Alves, Anabela Coelho

**Affiliations:** 1 Digital Human-Environment Interaction Labs (HEI-LAB), Lusófona University of Humanities and Technologies, Campo Grande 376, 1749-024 Lisbon, Portugal; 2 Portuguese Laboratory for Healthy Workplaces, Institute of Environmental Health, Lisbon University, 1400-185 Lisbon, Portugal; 3 Comprehensive Health Research Center (CHRC), University of Évora, 7004-516 Évora, Portugal; elisabete.alves@uevora.pt; 4 São João de Deus School of Nursing, University of Évora, 7000-801 Évora, Portugal; anabela.coelho@uevora.pt; 5 Global Health and Tropical Medicine, Instituto de Higiene e Medicina Tropical, Universidade NOVA de Lisboa, 1099-085 Lisbon, Portugal

**Keywords:** health personnel, psychological well-being, working conditions, healthy work environments

## Abstract

Background: Health professionals are at high risk of poor mental health and well-being. Faced with this challenge, healthcare organizations must be healthy and safe work environments. Objectives: This study aims to take an in-depth, systemic look at whether healthcare organizations are healthy workplaces. Methods: The study involved 2190 participants aged between 19 and 71 (M = 44.73, SD = 10.29) and data were collected in 12 public hospitals between November 2021 and December 2023. The study used the Ecosystems of Healthy Workplaces instrument, which consists of a total of 62 items organized into nine dimensions based on the Healthy Workplaces model proposed by the World Health Organization. Results: Most dimensions revealed a moderate risk in terms of whether they were healthy work environments, while the dimension that revealed a high risk was related to psychosocial risks at work in relation to well-being and mental health. A total of 87% of the professionals reported at least one symptom of burnout and 61.4% reported having all three symptoms of burnout. Additionally, 25.4% reported having been victims of harassment at work. When comparing the groups, we identified that the higher risk groups were namely women, generation Z and X professionals, doctors (compared to the different professional groups under analysis such as nurses, operational assistants, psychologists, administrators, senior technicians, and managers), professionals with chronic illnesses, and those who reported harassment at work. Conclusions: We conclude that the work environment must be understood ecologically, by analyzing the different systems and their relationships. This makes it possible to identify priority factors and groups for intervention.

## 1. Introduction

Worldwide, healthcare cost constraints, digital transformations, and workforce insta-bility, among other challenges, make healthcare professionals a vulnerable group within the healthcare system [1].

Healthcare professionals often face heavy workloads and stressful working conditions, which can have a negative impact on their physical and mental health [2,3]. Factors such as higher patient acuity, chronicity of care, lack of physical or psychological safety, perceived job security, and diminished team support [4,5], as well as physical and psychological demands such as constant exposure to illness and moral conflicts [6], contribute to the high prevalence of mental illness reported among health professionals [7]. This high risk to mental health and well-being [8,9,10] poses a major public health concern and threatens the delivery of quality care [11,12]. Therefore, it is crucial to identify and mitigate these work-related risk factors to protect healthcare workers’ mental health and well-being [13].

Currently, many countries are experiencing challenges in the education, employment, deployment, retention, and performance of their healthcare workforce [14]. This highlights the importance of engaging both international and national policy-makers to empower the role of mental health and occupational health professionals and promote the mental well-being of these workers [11,13].

Healthcare organizations must prioritize creating healthy and safe work environments by considering these challenges. Research indicates that fostering healthy work environments that prioritize the health and well-being of healthcare professionals [15] is crucial for improving recruitment and retention [14,16]. This contributes to better patient and societal outcomes and enhances organizational performance [17].

In the last decades, patient safety has become the main priority for healthcare organizations. This is primarily due to its association with a lower likelihood of significant complications, reduced clinical errors, and fewer adverse health events, all of which contribute to improved patient outcomes and increased efficiency and effectiveness [18,19].

Although a patient safety culture encourages collaboration and open communication among healthcare teams, so healthcare professionals feel comfortable reporting errors which leads to more effective and coordinated care [18], the relationship between organizational culture, healthcare professionals’ perceptions of psychological well-being, and patients’ experiences of care is complex and deserves greater attention and reflection [20]. Over time, low levels of patient safety culture have been shown to be related to a high prevalence of burnout across all healthcare professions [21]. The well-being of healthcare workers is crucial, not only for their health but also for maintaining high standards of patient safety and care quality. The healthcare relationship is bidirectional, which highlights the importance of caring for both participants (patients and healthcare professionals). In this context, if collecting and reporting patient experience data is crucial to assess the quality of care from patients’ perspectives (e.g.,: Patient Reported Experience Measures (PREMs) [22,23], we also should show same concern for the experience of the healthcare professionals and have systematic Professional Reported Experience Measure (ProfREMs) to encourage a more a holistic and multidisciplinary approach that effectively implements good practices for healthy and safe workplaces [22,23]. This involves engaging all stakeholders, including healthcare professionals, by collecting and analyzing their experiences and perspectives about their work environment and conditions.

According to the World Health Organisation [24], a healthy work environment is described as a place in which workers and managers collaborate to use a process of continuous improvement to protect and promote the safety, health, and well-being of all workers and the sustainability of the work environment, taking into account the following considerations which have been established on the basis of previously determined needs: (1) safety and health issues in the physical work environment; (2) safety, health, and well-being issues in the psychosocial work environment, including work organisation and organisational culture; (3) resources for personal health in the work environment, and (4) company involvement in the community to improve the health of workers, their families, and other community members. According to this conceptual model, a healthy work environment integrates central dimensions such as the organisation’s ethics and values, leadership commitment, and professional involvement, which are then integrated into four environments, namely the physical work environment, the psychosocial work environment, resources for personal health, and the organisation’s involvement in the community. The model also advocates promoting healthy working environments as an ongoing process that involves diagnosing, defining priorities, planning, implementing, and evaluating again in a process of continuous improvement that should involve all stakeholders inside and outside the organization [1].

Health professionals are among those with the greatest psychosocial risks at work, namely linked to the pace of work, cognitive and emotional demands, stress, and burnout [25].

This situation worsened with the COVID-19 pandemic and has recovered in the years since. Social and economic changes are also putting a strain on the public health system and organisations, including the ageing population, patients’ greater demands for healthcare, and the economic recession. The challenges facing human resource management in healthcare today are partly the result of these cumulative shocks [26].

The shortage of health professionals and the difficulties in their management models are common in many developed and developing countries. The determinants of this difficulty may increase in the coming years, posing challenges to the health system and requiring flexible and innovative responses [27].

In Portugal, these aspects have gained a lot of relevance as they have added to the great competitiveness of growing private health organizations that are increasingly attracting health professionals from the public sector, accumulating a nearly exclusive dedication to the private sector which thus abandons the public sector [28].

This highlights the importance of engaging both international and national policy-makers in empowering the role of mental health and occupational health professionals to promote the mental well-being of workers [11,13].

In Portugal, previous data concluded that professionals working in the hospital setting are vulnerable to psychosocial risk factors [28] and burnout [25], emphasizing the need to increase awareness on the work-related consequences for workers and their organizations, as well as the development of healthy environments that promote healthcare professionals’ physical and psychosocial well-being [29,30]. However, studies assessing the effect of psychosocial and organizational environments on health professionals’ health are lacking.

The aforementioned WHO model [24] can be operationalized through the EATS (Ecosystems of Healthy Work Environments) instrument [31]. The adaptation and application of the healthy work environments model specifically to healthcare organizations has not yet been carried out. Taking this into account, this study aims to take an in-depth and systemic look at healthcare organizations as healthy workplaces. We aim to study healthcare organizations as healthy work environments, according to the dimensions of the WHO Healthy Workplaces model [24]. We intend to deepen the association between a healthy work environment and the mental health and well-being of health professionals.

We want to answer the following research question: “What is the relationship between the work environment of the health organizations of the National Health Service (SNS) and the mental health and well-being of health professionals”, so we analyzed which dimensions of the work environment best explain the psychosocial risks in the work of health professionals.

## 2. Materials and Methods

### 2.1. Design and Participants

A national, cross-sectional, exploratory study with a convenience sample was carried out. A convenience sample was chosen, despite its limitations (developed within the limitations of the present study), for reasons of feasibility. It was not possible for the research team to gain access and obtain participation from all, or a random, stratified sample of healthcare organizations.

The majority of participants (75.4%) were female (*n* = 1101), aged between 19 and 71 years old, with a mean age of 44.73 and a standard deviation of 10.29.

### 2.2. Instruments

The instrument used comprises sociodemographic questions and the Healthy Workplace Ecosystems instrument (EATS) [31], developed based on the Healthy Workplaces model proposed by the World Health Organization [24].

In this study, only eight scales from the EATS were used: (1) culture and values (8 items, α = 0.91) (CE), leadership commitment (LC) (6 items, α = 0.95), psychosocial risks related to mental health (PR mental health), physical environment (PE) (5 items, α = 0.92), telework (3 items, α = 0.82), community involvement (CI) (12 items, α = 0.90); health resources (HRs) (4 items, α = 0.83), psychosocial risks of work (PRW) (12 items, α = 0.91), engagement (4 items, α = 0.89), and performance (3 items, α = 0.86). All questions have a 5-point Likert-type scale. We excluded the telework scale because it does not have great expression in health professionals.

The 4-item version of the Stress Perception Scale (EPS) [32,33] was used to assess the degree to which an individual evaluates their life situations as stressful (α = 0.77).

The instrument included a total of 73 items/questions (7 sociodemographic items, 62 items from the Healthy Workplace Ecosystems instrument (EATS), and 4 items from the Stress Perception Scale).

### 2.3. Procedure

Permission to conduct this study was requested and approved by the Ethics Committee of the hospital and Professor Doutor Fernando Fonseca (18 March 2021/No 031/2021).

The study was developed between November 2021 and December 2023, in the post-COVID-19 pandemic context. Several health organizations from the National Health Service (public sector), spread throughout the country, were invited to participate in this study. In Portugal, there are four types of health organizations: public health organizations, private health organizations, social health organizations, and public health organizations with private management. The management and financing characteristics are very different between the four types. For the present study, we only chose to include public organizations to allow for a more adequate comparison. A meeting was held with the administrations of healthcare organizations to explain the study and clarify issues. A total of 12 health organizations agreed to participate and were included in the study, 6 organizations located in the north of Portugal and 6 organizations located in the south of Portugal. The response rate ranged from 10% (minimum required) to 20%. The questions in the questionnaire were mandatory for the final submission of the questionnaire; that is, all questionnaires submitted were fully completed and missing values did not apply.

As an inclusion criterion, participants had to be healthcare professionals (doctors, nurses, operational assistants, psychologists, administrators, senior technicians, and managers) from the healthcare organization. Professionals who worked in the health organization through external hiring (external company or temporary employment company) or the provision of services (green receipts) were excluded, and this information was included in the message inviting them to participate in the study.

Those organizations who agreed to participate received an online version of the instrument. A contact person from the organization spread the link internally among their workers from all professional categories through the institutional email. All professionals from each organization were invited to participate, but the sample was by convenience because just few organizations agreed to participate in the study.

The first page of the online version of the instrument included an explanation of the study, the contact details of the researchers for further queries and details, and information on confidentiality and anonymity. The participants only had access to the instrument items after they voluntarily signed the informed consent form. At the end of the data collection, each health organization involved received an individual report with the organization’s aggregate results, risk index in the different dimensions, and recommendations for promoting a healthier working environment.

## 3. Results

The majority of participants (43.3%) were from Generation X (*n* = 638). In terms of the professional category, the participants were mostly (34.4%) nurses (*n* = 506). Regarding chronic illness status, the majority of the participants (64.2%) did not have one (*n* = 945) (Table 1).

Table 2 and Table 3 show the comparison of groups related to each of the variables of gender, chronic illness, generation, and professional category. Statistically significant differences in CE, CI, and performance were observed. In other words, women showed more positive values in CE, engagement, and performance than men. Values for each of the dimensions are as follows: CE (men—M = 2.88, SD = 0.90; women—M = 3.00, SD = 0.88, *p* = 0.031), LC (men—M = 2.63, SD = 1.06; women—M = 2.73, SD = 1.05, *p* = −140 (n.s)), PR mental health (men—M = 3.37, SD = 0.98; women—M = 3.39, SD = 1.00, *p* = 0.081 (n.s)), PE (men—M = 3.08, SD = 1.14; women—M = 2.97, SD = 1.07, *p* = 0.100 (n.s)), telework (men—M = 3.34, SD = 0.96; women—M = 3.16, SD = 1.04, *p* = 0.064 (n.s)), CI (men—M = 3.32, SD = 0.71, women—M = 3.35, SD = 0.69, *p* = 0.446 (n.s)), HRs (men—M = 2.40, SD = 0.96; women—M = 2.39, SD = 0.93, *p* = 0.801 (n.s)), LS (men—M = 2.59, SD = 0.75; women—M = 2.65; SD = 0.76, *p* = 0.220 (n.s)), PWE (men—M = 2.95, SD = 0.94, women—M = 2.99, SD = 0.99, *p* = 0.470 (n.s)), engagement (men—M = 3.19, SD = 0.90; women—M = 3.38, SD = 0.89, *p* < 0.001), and performance (men—M = 4.19, SD = 0.72, women—M = 4.35, SD = 0.63, *p* < 0.001).

Regarding chronic illness, statistically significant differences were observed in leadership commitment (LC), PR mental health, physical environment (PE), health resources (HRs), and labor stress (LS). People who did not suffer from a chronic illness showed more positive values in LC, PE, and HRs. On the other hand, people who had a chronic illness showed higher scores for PR mental health and WS. Each of the dimensions for this variable are as follows: CE (no CD—M = 2.99, SD = 0.88; has CD—M = 2.92, SD = 0.91, *p* = 0.150 (n.s)), LC (no CD—M = 2.74, SD = 1.04; has CD—M = 2.61; SD = 1.08, *p* = 0.019), PR mental health (no CD—M = 3.32, SD = 0.99; has CD—M = 3.52, SD = 0.99, *p* < 0.001), PE (does not have CD—M = 3.08, SD = 1.08; has CD—M = 2.84, SD = 1.10, *p* < 0.001), telework (does not have CD—M = 3.23, SD = 1.03; has CD—M = 3.16, SD = 1.01, *p* = 0.452 (n.s)), community involvement (CI) (no CD—M = 3.36, SD = 0.79; has CD—M = 3.29; SD = 0.71, *p* = 0.055 (n.s)), HRs (no CD—M = 2.43, SD = 0.94; has CD—M = 2.32, SD = 0.93, *p* = 0.020), stress (no CD—M = 2.58, SD = 0.74; has CD—M = 2.74; SD = 0.79, *p* < 0.001), psychosocial work environment (PWE) (no DC—M = 3.01, SD = 0.93; has DC—M = 2.92, SD = 0.98, *p* = 0.076 (n.s)), engagement (no DC—M = 3.35, SD = 0.89; has CD—M = 3.30; SD = 0.92, *p* = 0.294 (n.s)), and performance (does not have CD—M = 4.29, SD = 0.66; has CD—M = 4.34, SD = 0.63, *p* = 0.254 (n.s)).

As for generation, statistically significant differences were observed for most of the variables, except for PE, telework, and PWE. Generation X had a high value for telework. Generation Y had higher values for PR mental health. Generation Z had higher values for CE, LC, PE, CI, HR, stress management, and PWE. In turn, the Baby boomer generation shows positive values in engagement and performance (Table 3).

As for the professional category, statistically significant differences were observed for all variables except performance. Doctors showed higher scores for mental health and stress management. Psychologists showed higher values for telework, PWE, and engagement. Administrators scored higher on CE, LC, and HR. Managers showed high values for PWE and CI (Table 4).

To examine the relationship between PR mental health and age, sex, chronic illness, culture and values, leadership commitment, physical work environment, community involvement, health resources, psychosocial work environment, engagement, performance, and work stress, a linear regression was conducted.

The regression model was statistically significant, F (12) = 71.531, *p* < 0.001, indicating that the independent variables explained a significant portion of the variance in the dependent variable. The adjusted R squared value was 0.367, suggesting that approximately 36.7% of the variance in PR mental health can be explained by the predictors in the model (Table 5).

The correlations were mostly significant. A positive, strong, and significant association was observed between CE and LC (r = 0.868, *p* < 0.001). In addition, moderate, positive, and significant associations were observed between LC and PWE (r = 0.673, *p* < 0.001), CE and PWE (r = 0.658, *p* < 0.001), PWE and engagement (r = 0.635, *p* < 0.001), CE and engagement (r = 0.564, *p* < 0.001), and LC and engagement (r = 0.531, *p* < 0.001) (Table 6).

## 4. Discussion

The results of the study allow us to verify that the mental health of health professionals can be explained by several dimensions of the ecosystem in health organizations.

Psychosocial risks of work related to mental health are best explained by work stress, followed by perceived performance. The dimensions of a healthy workplace, related to the organization’s culture, leadership commitment, psychosocial environment, and health resources, also help to explain the mental health of health professionals.

In a potentially stressful context such as health organizations, the perception of stress management skills is a factor that has a high impact on the mental health of professionals. Professionals who report that they feel unable to control the things that are important in life, who often feel that difficulties have accumulated to the point of not being able to overcome them, and who do not feel confident in their abilities to overcome problems are the ones who reveal worse mental health indicators [14,17].

Professionals who have a more favorable perception of the culture of health organizations, namely those that consider that the organization focuses on the well-being of workers and has policies and strategies to promote it, reveal more positive mental health. The commitment of leadership, namely those that consider that the leadership sees the well-being of employees as its priority, as well as a safer and more confident psychosocial environment reflected in the perception that professionals are respected and treated fairly in the workplace, also contributes to the well-being of professionals.

The proactivity of the health organization in promoting resources for the health of professionals, for example providing workers with actions and programs to adopt healthy behaviors (smoking cessation, nutrition, stress management, healthy sleep habits, etc.), is a factor that facilitates the adoption of health behaviors [1,13].

The current internal and external conditions of health organizations make the reality of these professionals very challenging [2,3].

Finally, it is the professionals with the best perception of performance who report greater psychosocial risks related to mental health at work.

An in-depth and comparative study was also carried out, comparing men and women, different generations, professional areas, and health professionals with and without chronic disease.

The gender comparison revealed that women are the ones who are most satisfied with organizational culture, who report that they are involved, and who reveal better performance. Several studies have revealed that women report feeling more satisfied at work [34]; on the other hand, this leads us to hypothesize that this aspect is associated with less assertiveness and demand from women in the context of work.

Comparing the four generations currently in the labor market, we found that it is the younger generation (Generation Z) that reveal more psychosocial risks in work related to mental health and less stress management skills. On the other hand, it is the older generations that reveal greater perceptions of involvement and performance. Studies carried out with young health professionals, especially physicians in the years of medical internship, reveal greater difficulty in terms of mental health, possibly associated with the demands of the work (emotional, physical, and rhythm) and the great responsibility in decision-making on subjects without much professional experience [35].

Comparing the different types of professionals in a health organization showed that it is doctors and nurses who reveal more psychosocial risks related to mental health at work and less stress management skills. Doctors and nurses suffered from more psychological challenges in their day-to-day professional life in the hospital context [36]. This was also exacerbated by the COVID-19 pandemic [37], and not much has been done to mitigate this impact or promote the mental health of these professionals.

Comparing professionals with different health conditions, we found that professionals with chronic illness, comprising more than a third, were the ones who reported more negative perceptions of leadership involvement, more psychosocial risks of work related to mental health, and less stress management skills. On the other hand, they also reported having less health resources provided by the health organizations where they work. This result is related to the greater mental health risk revealed by professionals with chronic illnesses [38].

The present study contributes to the assessment of the adaptability of the WHO Healthy Workplaces model [24] in healthcare organizations, allowing us to characterize the different dimensions of the model, the relationships between them, and the way they help to explain the mental health of healthcare professionals.

The study has some limitations, namely the fact it was a cross-sectional study that did not allow causality to be assumed. Although we had a national and illustrative sample of several health organizations, a convenience sample does not allow for generalization of the results and conclusions. A convenience sample has several limitations that can affect the validity of the results: (1) selection bias, as the sample is made up of individuals who are readily available and it may not adequately represent the target population, leading to biased results; (2) limited generalization, as the results obtained from a convenience sample may not be applicable to other contexts or populations as the sample may not reflect the diversity of the population; (3) lack of control over variables, as it is more difficult to control external variables that may influence the results, since the sample is not chosen randomly; (4) reliability problems, which can result in less reliable and reproducible data; (5) a tendency towards homogeneous groups, as the sample may be made up of individuals with similar characteristics which limits the variability and depth of the analysis, and (6) difficulties in identifying causality, as the relationship between variables may be more difficult to establish since the sample may not have been selected for a specific purpose. These limitations are important to consider when interpreting the results of studies using convenience samples. However, we consider this sample to be illustrative of the study population because it included many health organizations from different areas (large urban centers and smaller cities) and regions of Portugal, including hospitals of different sizes with different types of population served (patients). The sample size is also reasonable, as we have 150,000 health professionals in Portugal, and when calculating the sample size we found that for a 95% confidence level and 5% margin of error we would need 384 subjects, and our sample is substantially higher.

## 5. Conclusions

We conclude that healthy work environments have different dimensions related to the mental health of health professionals.

Occupational stress and performance perception influence mental health, which leads to a need to reduce stress-related risks, promote stress management skills, and promote greater balance and fairness in the demand for performance among health professionals, especially in relation to emotional and physical demands, the pace of work, and recognition and appreciation [21,25].

Groups that were at even higher risk for poor mental health included younger professionals (usually in medical internships), doctors and nurses, and health professionals with chronic illnesses who need specific and robust prevention and recovery intervention.

How healthcare organizations can have a healthy work environment must be understood in an integral and systemic way. An organization’s culture must be health-promoting, with leaders that are more humanized and that have specific skills to promote better communication, greater appreciation, justice, and recognition towards professionals. There is a need to develop activities that promote well-being and health in the work context, as well as promoting better communication and articulation between professionals at different levels of the organizational structure. Mental health intervention, burnout prevention, and the development of conflict and stress management strategies are priority areas for intervention. It is pertinent that leaders (managers) practice more humanized ways of managing work teams, not solely focusingon “governance by numbers” which contributes to the degradation of work environments and workers’ health. Healthy work environments and healthier healthcare professionals contribute to more appropriate healthcare delivery and promote greater satisfaction and, indirectly, better patient health [23,39].

The results culminate in the following recommendations for promoting healthy work environments.

(1) An inclusive work culture: Healthy work environments have a clear communication culture and expectations for workers, and are realistic about goals and working conditions. They adopt a balance between professional and personal life, learn from mistakes in a productive way, and encourage participation;

(2) A safe environment: Healthy work environments ensure that workers do not encounter unnecessary dangers while carrying out their duties. They ensure that work areas meet regulations, which must be in line with realistic needs for adequate performance, that is, with “real work”, and have safety protocols and promote safe practices;

(3) Competent leadership: A healthy work environment includes empathetic, assertive, fair, transparent leadership, who are on the side of the professional, and who reflect psychological safety, equity and equality in a continuous process of support, encouragement, and autonomy;

(4) Effective interpersonal relationships: Interpersonal relationships between colleagues are very important for the perception of a healthy work environment, teamwork, mutual help, mentoring, and supervision, with healthy informal moments of coexistence. It is important to promote the spirit of cooperation so that it is possible to build cohesive and protective work collectives;

(5) Professional support and involvement in a developmental context: A healthy work environment should allow professionals to have opportunities for development, without encouraging unnecessary competition. A joint process of goal setting, skills development and training, incentives and appreciation can create more value for the organization and more opportunities for its future.

For future studies, a more specific instrument could be used to assess burnout in health professionals, and a qualitative study could be developed using interviews and focus groups to deepen the results obtained and identify strategies for the promotion of healthier, safer, and more sustainable work environments for health professionals. It would also be interesting to study healthy work environments by comparing the different types of health organizations that exist in Portugal, namely public health organizations, private health organizations, social health organizations, and privately managed public health organizations. This would allow for an understanding of how different forms of management and financing could influence healthy work environments in their different dimensions. Currently, the National Health Service is reorganizing its health organizations into 39 health groups that integrate hospitals and health centers in different areas of the country. We intend to contribute to the evaluation and monitoring of this change in the medium and long term through assessment with the EATS instrument.

We focused this study on the promotion of global health (bio-psycho-social) and healthy lifestyle. Healthy work environments encourage workers to maintain good mental health, nutrition, physical exercise, consumption, sleep habits, and stress management skills, allowing them to perform better and more reliably. Finally, it should be noted that the promotion of healthy work environments is a continuous process of evaluation, intervention, and monitoring that must involve all stakeholders and be accompanied by a multidisciplinary team, with doctors, nurses, and psychologists among other professionals, as a necessity to to ensure healthy and safe working environments.

## Figures and Tables

**Table 1:** Sociodemographic characteristics of the sample (*n* = 1475).

Variables		% or M ± SD
Sex (*n* = 1475)	Female	75.4 (1101)
	Male	24.6 (360)
Age (*n* = 1473)		Min = 19; Max = 71 44.73 ± 10.29
Generation * (*n* = 1475)	Z	2.3 (34)
	Y (Millennial)	42.8 (630)
	X	43.3 (638)
	Baby boomer	11.6 (171)
Professional category (*n* = 1475)	Doctor	20.8 (306)
	Nurse	34.4 (506)
	Assistant	20.1 (296)
	Psychologist	3.7 (55)
	Administrative	2.0 (30)
	Superior Technician	17.2 (253)
	Manager	1.8 (26)
Chronic illness (*n* = 1473)	No	64.2 (945)
	Yes	35.8 (528

* Baby boomers, born between 1946 and 1964; Generation X, born between 1965 and 1980; Millennials or Generation Y, born between 1981 and 1996; Generation Z: born between 1997 and 2010 (source: U.S. Census Bureau).

**Table 2:** Descriptive statistics and comparison analysis according to sex and chronic illness.

*	Descriptive Statistics				Significance Tests and Effect Size
	$\bar{x}$	SD	$\bar{x}$	SD	
	Male		Female		
CV	2.88	0.90	3.00	0.88	t (1459) = 2.163, *p* = 0.031; d = 0.89
LC	2.63	1.06	2.73	1.05	t (1459) = 1.478, *p* = 0.140; d = 1.05
PR mental health	3.37	0.98	3.39	1.00	t (1459) = 0.252, *p* = 0.081; d = 0.99
PE	3.08	1.14	2.97	1.07	t (579.313) = −1.648, *p* = 0.100; d = 1.09
Telework	3.34	0.96	3.16	1.04	t (575) = −1.855, *p* = 0.064; d = 1.02
CI	3.32	0.71	3.35	0.69	t (1459 = 0.763, *p* = 0.446; d = 0.70
HRs	2.40	0.96	2.39	0.93	t (1459) = −0.171, *p* = 0.801; d = 0.94
Work stress	2.59	0.75	2.65	0.76	t (1459) = 1.227, *p* = 0.220; d = 0.76
PRW	2.95	0.94	2.99	0.95	t (1459) = 0.723, *p* = 0.470; d = 0.95
Engagement	3.19	0.90	3.38	0.89	t (604.062) = 3.478, *p* < 0.001; d = 0.89
Performance	4.19	0.72	4.35	0.63	t (1459) = 4.121, *p* < 0.001; d = 0.65
	Does not have a CD		Has a CD		
CV	2.99	0.88	2.92	0.91	t (1471) = 1.441, *p* = 0.150
LC	2.74	1.04	2.61	1.08	t (1471) = 2.350, *p* = 0.019
PR mental health	3.32	0.99	3.52	0.99	t (1471) = −3.783, *p* < 0.001
PE	3.08	1.08	2.84	1.10	t (1471) = 4.031, *p* < 0.001
Telework	3.23	1.03	3.16	1.01	t (580) = 0.753, *p* = 0.452
CI	3.36	0.69	3.29	0.71	t (1471) = 1.917, *p* = 0.055
HRs	2.43	0.94	2.3	0.93	t (1471) = 2.337, *p* = 0.020
Work stress	2.58	0.74	2.74	0.79	t (1471) = −3.871, *p* < 0.001
PRW	3.01	0.93	2.92	0.98	t (1471) = 1.778, *p* = 0.076
Engagement	3.35	0.89	3.30	0.92	t (1471) = 1.051, *p* = 0.294
Performance	4.29	0.66	4.34	0.63	t (1471) = −1.141, *p* = 0.254

* Culture and values, CV; leadership commitment (LC); psychosocial risks related to mental health, PR mental health; physical environment, PE; community involvement, CI; health resources, HRs; psychosocial risks of work, PRW.

**Table 3:** Descriptive statistics and comparison analysis according to generations.

	$\bar{x}$ (SD)	$\bar{x}$ (SD)	$\bar{x}$ (SD)	$\bar{x}$ (SD)	Significance Test and Effect Size
	Generation Z *	Generation Y *	Generation X *	Baby boomer *	
CE	3.37 (0.96)	2.92 (0.87)	2.99 (0.91)	2.96 (0.85)	F (3) = 2.963, *p* = 0.031, η = 0.006
LC	3.11 (1.18)	2.63 (1.03)	2.74 (1.09)	2.66 (0.98)	F (3) = 3.045, *p* = 0.028; η = 0.006
PR mental health	3.16 (1.17)	3.49 (1.00)	3.35 (0.97)	3.22 (0.98)	F (3) = 4.717, *p* = 0.003; η = 0.010
PE	3.13 (1.11)	2.96 (1.09)	3.00 (1.07)	3.11 (1.19)	F (3) = 1.048, *p* = 0.370; η = 0.002
Telework	2.73 (1.10)	3.18 (1.02)	3.24 (1.01)	3.24 (1.14)	F (3) = 1.261, *p* = 0.287; η = 0.007
CI	3.52 (0.68)	3.25 (0.69)	3.40 (0.70)	3.42 (0.69)	F (3) = 6.763, *p* < 0.001; η = 0.014
HRs	2.81 (1.08)	2.29 (0.91)	2.46 (0.95)	2.41 (0.91)	F (3) = 6.065, *p* < 0.001; η = 0.012
Work Stress	2.88 (0.84)	2.75 (0.75)	2.57 (0.76)	2.42 (0.69)	F (3) = 11.977, *p* < 0.001; η = 0.024
PWE	3.25 (1.05)	2.97 (0.92)	2.98 (0.98)	2.90 (0.91)	F (3) = 1.233, *p* = −296; η = 0.003
Engagement	3.35 (0.98)	3.22 (0.87)	3.40 (0.92)	3.48 (0.85)	F (3) = 6.126, *p* < 0.001; η = 0.012
Performance	4.04 (0.94)	4.27 (0.64)	4.34 (0.65)	4.40 (0.61)	F (3) = 4.545, *p* = 0.004; η = 0.009

* Baby boomers, born between 1946 and 1964; Generation X, born between 1965 and 1980; Millennials or Generation Y, born between 1981 and 1996; Generation Z, born between 1997 and 2010 (source: U.S. Census Bureau).

**Table 4:** Comparison analysis according to professional category.

	$\bar{x}$ (SD)	$\bar{x}$ (SD)	$\bar{x}$ (SD)	$\bar{x}$ (SD)	$\bar{x}$ (SD)	$\bar{x}$ (SD)	$\bar{x}$ (SD)	Significance Test and Effect Size
	Doctor	Nurse	Assistant	Psychologist	Administrative	Superior technician	Manager	
CE	2.92 (0.94)	2.91(0.84)	2.99 (0.97)	3.37 (0.88)	3.50 (0.64)	2.93 (0.81)	3.13 (0.99)	F (6) = 4.436, *p* < 0.001; η = 0.018
LC	2.63 (1.10)	2.59 (1.00)	2.76 (1.11)	3.20 (1.06)	3.35 (0.77)	2.69 (1.00)	3.05 (1.18)	F (6) = 5.866, *p* < 0.001; η = 0.023
PR mental health	3.64 (0.99)	3.48 (0.91)	3.13 (1.07)	3.07 (0.92)	3.15 (0.72)	3.32 (1.01)	3.29 (0.97)	F (6) = 9.358, *p* < 0.001; η = 0.037
PE	2.81 (1.15)	2.95 (1.08)	3.05 (1.11)	3.07 (1.01)	3.48 (0.93)	3.13 (1.04)	3.50 (0.84)	F (6) = 4.278, *p* < 0.001; η = 0.017
Telework	3.05 (1.03)	3.01 (0.98)	3.14 (1.02)	3.67 (1.12)	3.61 (0.99)	3.58 (0.95)	3.17 (1.16)	F (6) = 5.341, *p* < 0.001; η = 0.053
CI	3.23 (0.70	3.32 (0.72)	3.36 (0.68)	3.28 (0.73)	3.51 (0.54)	3.44 (0.67)	3.62 (0.63)	F (6) = 3.367, *p* = 0.003; η = 0.014
HRs	2.01 (0.82)	2.21 (0.87)	2.64 (0.91)	2.75 (1.02)	3.18 (0.82)	2.68 (0.94)	3.17 (1.02)	F (6) = 30.041, *p* < 0.001; η = 0.110
Work stress	2.72 (0.80)	2.61 (0.74)	2.67 (0.76)	2.33 (0.72)	2.49 (0.66)	2.66 (0.76)	2.47 (0.71)	F (6) = 2.735, *p* = 0.012; η = 0.011
PWE	3.06 (0.98)	2.92 (0.86)	2.82 (0.97)	3.59 (0.87)	3.55 (0.81)	2.94 (0.95)	3.21 (1.10)	F (6) = 8.393, *p* < 0.001; η = 0.033
Engagement	3.31(0.91)	3.28 (0.87)	3.28 (0.91)	3.92 (0.83)	3.49 (0.76)	3.35 (0.89)	3.48 (1.03)	F (6) = 4.790, *p* < 0.001; η = 0.019
Performance	4.32 (0.54	4.27 (0.62)	4.29 (0.88)	4.38 (0.50)	4.47 (0.48)	4.37 (0.54)	4.41 (0.69)	F (6) = 1.356, *p* = 0.229; η = 0.006

**Table 5:** Regression model.

		Non-Standard Coefficients		Standardized Coefficients		
		B	Standard Error	β	t	*p*
PR mental health	(Constant)	1.24	0.24		5.18	<0.001
	Age	−0.00	0.00	−0.00	−1.01	>0.05
	Sex (2—Male)	0.08	0.05	0.03	1.53	>0.05
	Chronic illness	0.06	0.05	0.03	1.28	>0.05
	CE	0.18	0.05	0.16	3.63	<0.001
	LC	−0.16	0.04	−0.17	−3.85	<0.001
	PE	−0.04	0.02	−0.04	−1.73	>0.05
	CI	−0.01	0.04	−0.01	−0.20	>0.05
	HR	−0.12	0.03	−0.12	−4.70	<0.001
	PWE	−0.16	0.03	−0.15	−4.61	<0.001
	Engagement	−0.09	0.04	−0.08	−2.61	<0.05
	Performance	0.41	0.04	0.27	11.29	<0.001
	Work stress	0.56	0.03	0.43	18.55	<0.001

**Table 6:** Correlations.

	CE	LC	PR Mental Health	PE	Telework	CI	HR	Labor Stress	PWE	Engagement	Performance
CE		0.862 *	−0.273 *	0.277 *	0.037	0.363 *	0.442 *	−0.230 *	0.658 *	0.564 *	0.087 *
LC			−0.324 *	0.269 *	0.015	0.357 *	0.443 *	−0.217 *	0.673 *	0.531 *	0.045
PR Mental health				−0.188 *	−0.006	−0.172 *	−0.290 *	0.487 *	−0.346 *	−0.283 *	0.124 *
PE					0.207 *	0.435 *	0.289 *	−0.148 *	0.240 *	0.247 *	0.040
Telework						0.213 *	0.107 *	−0.091 *	0.002	0.032	0.027
CI							0.347	−0.156 *	0.289 *	0.296 *	0.111 *
HR								−0.179 *	0.396 *	0.335 *	0.034
Labor Stress									−0.306 *	−0375 *	−0.180 *
PWE										0.635 *	0.195 *
Engagement											0.410 *
Performance											

* *p* < 0.001.

## Data Availability

The data presented in this study are available on request from the corresponding author. The data are not publicly available due to the ownership of the institutions where the study was conducted.
